# Characterization of ADGRG6 as a potential molecular oncotarget of pancreatic cancer

**DOI:** 10.1038/s41419-026-08766-2

**Published:** 2026-05-06

**Authors:** Qian-hui Gu, Xiao-ren Zhu, Jian-zhuo Jiang, Na Liu, An-qi Jin, Yan Zhang, Jing-jing Lu, Ping Li, Zhen-yu Ye, Yuan-yuan Liu, Min-bin Chen

**Affiliations:** 1 Department of Radiotherapy and Oncology, Affiliated Kunshan Hospital of Jiangsu University, Kunshan, China; 2 Department of Radiotherapy and Oncology, SuzhouMedical College of Soochow University, The First People’s Hospital of Kunshan, Kunshan, China; 3 Stem Cell Institute, Department of Development and Regeneration, Katholieke Universiteit (KU) Leuven, Leuven, Belgium; 4 Clinical Research and Lab Center, Affiliated Kunshan Hospital of Jiangsu University, Kunshan, China; 5 Department of Laboratory Medicine, Taicang Loujiang New City Hospital (Ruijin Hospital Taicang), Taicang, China; 6 The BioBank, Affiliated Kunshan Hospital of Jiangsu University, Kunshan, China; 7 Department of General Surgery, The Second Affiliated Hospital of Soochow University, Suzhou, China

**Keywords:** Pancreatic cancer, Tumour biomarkers

## Abstract

Identifying novel therapeutic targets for pancreatic cancer (PC) is crucial for improving patient outcomes. This study identified the functions, expression, and associated mechanisms of adhesion G protein-coupled receptor G6 (ADGRG6/ GPR126) in PC. Bioinformatics analyses revealed substantial upregulation of ADGRG6 in human PC, correlating with poor survival rates and advanced tumor stages. Elevated ADGRG6 expression has been observed in human PC tissues and cell lines. Targeted depletion of ADGRG6 *via* the CRISPR/Cas9 knockout (KO) or lentiviral shRNA technology in established and primary PC cells (priPC-1) resulted in a substantial decrease in cell cycle progression, cell proliferation, viability, as well as reduced migratory and invasive capabilities. Conversely, ADGRG6 overexpression further enhanced the malignant behavior of PC cells. Mechanistically, ADGRG6 is crucial for Akt-mTOR cascade activation. ADGRG6 depletion markedly decreased Akt, S6, and 4E-BP1 phosphorylation. Constitutively active mutant Akt1 (S473D, caAkt1) reversed the anti-proliferative and anti-migratory effects of ADGRG65 shRNA and restored Akt-mTOR phosphorylation. Further analysis revealed that ADGRG6-driven Akt-mTOR activation is mediated by G protein inhibitory subunit 3 (Gαi3). ADGRG6 shRNA significantly inhibited subcutaneous PC xenograft growth in mice, accompanied by reduced Akt-mTOR signaling activation. In contrast, ADGRG6 overexpression promotes xenograft growth. Together, these findings establish ADGRG6 as a critical mediator of PC progression via Gαi3-dependent activation of the Akt-mTOR axis. Targeting ADGRG6 is a promising therapeutic strategy for combating PC.

## Introduction

Pancreatic cancer (PC) is a highly lethal malignancy with a five-year survival rate of less than 10% [1, 2]. Recurrence rates in patients with early-stage PC range from 50 to 70% within one year following surgery [3]. The poor prognosis associated with PC is primarily due to late diagnosis, distant metastases, and intrinsic resistance to conventional treatment modalities [4]. Chemotherapy regimens incorporating irinotecan liposomes or gemcitabine are the standard first-line treatments for advanced PC [5–7]. However, their clinical utility is significantly hindered by poor patient tolerance, as well as the systemic and multifocal characteristics of advanced disease, which collectively diminish the efficacy of systemic intravenous chemotherapy [8, 9]. Furthermore, the therapeutic outcomes of immunotherapy and radiotherapy for PC remain unsatisfactory [2]. Therefore, a deeper insight into the molecular mechanisms underlying PC progression is crucial for identifying and developing effective targeted treatments.

G-protein-coupled receptors (GPCRs), a fundamental and highly diverse superfamily of membrane-bound proteins mediating signal transduction, have been increasingly recognized for their pivotal roles in tumorigenesis and cancer progression [10, 11]. Aberrant expression of specific GPCRs is observed across a range of cancer types, where these dysregulated receptors are implicated in key processes, such as cell transformation, proliferation, angiogenesis, metastasis, drug resistance, and apoptosis [12, 13]. Importantly, GPCRs and their associated signaling pathways are highly druggable targets, with over 40% of currently approved pharmaceuticals acting on this receptor family [14, 15]. Targeting GPCRs holds significant promise for tumor eradication. Particularly in PC, certain GPCRs, such as G protein-coupled receptor 5 A (GPCR5A) expressed in PC cells and G protein-coupled receptor 68 (GPR68) detected on cancer-associated fibroblasts (CAFs) in the tumor microenvironment, serve as biomarkers and potential drug targets for cancer therapy [16, 17].

Adhesion G-protein-coupled receptors (aGPCRs), which constitute the second largest GPCR subfamily, are ubiquitously expressed across multiple tissues and organs and are recognized as critical regulators of diverse physiological and pathological processes [18]. Within this subfamily, ADGRG6, (also known as GPR126), has emerged as a critical regulator of cell adhesion, migration, paracrine signaling, and disease progression [19]. Genetic alterations in ADGRG6, including copy number variations and mutations, have been identified in multiple cancers, correlating with tumor progression and patient outcomes [20–23]. Additionally, ADGRG6 contributes significantly to endothelial cell recruitment and tumor angiogenesis regulation [24, 25]. In lung cancer, ADGRG6 promotes malignant transformation via oncogenic gene fusions [26], and its oncogenic potential has also been demonstrated in colorectal cancer and acute myeloid leukemia, where it enhances tumor cell proliferation and disease progression [27, 28]. This study is designed to explore the expression and functional roles of ADGRG6 in PC.

## Materials and methods

### Antibodies

The primary antibodies included antibodies against mTOR (1:1000; #2983), phospho-mTOR (#5536), phospho-Akt Ser-473 (#4060), Akt (#4685), S6 (#2217), phospho-S6 (#4858), 4E-BP1 (#9644), phospho-4E-BP1 (#2855), E-Cadherin (#3195) (all acquired from CST and diluted at 1:1000), and Gαi3 (1:200; ab154024), Vimentin (1:1000; #92547), N-Cadherin (1:1000; #245117), and β-actin (1:2000) (all obtained from Abcam), as well as ADGRG6 (1:1000; orb339171, Biorbyt, Shanghai, China).

### Tissue microarray and cells

The immortalized cell lines CFPAC-1, BxPC-3, PATU-8988, and hTERT-HPNE were sourced from the Institute of Biochemistry and Cell Biology, Chinese Academy of Sciences (Shanghai, China) and grown in high-glucose Dulbecco’s Modified Eagle Medium containing 10% fetal bovine serum and 1% penicillin-streptomycin. After obtaining informed consent, Primary PC cells (designated as “priPC-1” and “priPC-2”), were isolated from tumor specimens obtained from two male patients (aged 72 and 58 years) who had received a pathological diagnosis of pancreatic ductal adenocarcinoma at the Department of Oncology, Kunshan First People’s Hospital. Detailed procedures for isolation, establishment, and culture of primary PC (priPC) cells were conducted according to described methods previously [29]. The primary PC cell lines (“priPC-1、priPC-2”) exhibits classic molecular characteristics, including an activating KRAS mutation, a loss-of-function mutation in TP53. The primary cell line priPC-1 also exhibits somatic mutations in BRCA1, GRIN2A, and SPTA1. A human PC tissue microarray was acquired and evaluated by Shanghai Outdo Biotech Co. Ltd (Shanghai, China). All experiments involving human clinical samples were conformed to the ethical guidelines outlined in the Declaration of Helsinki and approved by the Ethics Committee of Kunshan Hospital Affiliated with Jiangsu University (Number: 2025-03-007-H00-K01).

### Data analysis for single-cell RNA sequencing (scRNA-seq)

The scRNA-seq data analysis was conducted using R (version 4.1.3) and the Seurat package (version 4.2.0). Cell-by-gene count matrices for each sample were processed using the “CreateSeuratObject” function with a stringent inclusion criterion of a minimum of three cells per gene. Cells exhibiting a high proportion of mitochondrial or erythrocyte gene expression ( >15%) or extreme gene counts ( <100 or >7000), indicative of compromised cell integrity, were excluded. Following quality control, 10,664 cells were selected for subsequent analyses. NormalizeData and ScaleData functions were employed for data normalization and scaling, respectively. The FindVariableFeatures function was utilized to identify significantly variable genes, which were then employed for principal component analysis (PCA) for dimensionality reduction. Optimal principal component numbers for further analysis were determined using the “JackStraw” and “ElbowPlot” functions. Dimensionality reduction was subsequently performed using t-distributed stochastic neighbor embedding (t-SNE) and Uniform Manifold Approximation and Projection (UMAP). Clustering of cells was executed with the “FindClusters” function to identify distinct cellular populations. Differentially expressed genes (DEGs) between tumor and normal cell clusters were determined *via* the “FindMarkers” function, with significance thresholds of log fold changes >1 and *p-values* < *0.05*.

### Cellular functional studies and gene/protein detection

Comprehensive protocols for various cellular functional assays, such as colony formation, CCK-8 cell viability analysis, “Matrigel Transwell” invasion assays, EdU staining for cell proliferation, “Transwell” migration assays, and analysis of the cell cycle using PI staining followed by flow cytometry, have been extensively detailed in our previous publications [30, 31]. Additionally, detailed methodologies for quantitative real-time PCR (qRT-PCR) and western blotting have also been thoroughly documented in earlier studies [31].

### Gene silencing

Two distinct ADGRG6 shRNA sequences, namely shADGRG6-s1 (targeting 5’-CCTCACTTTCATCAGCTATAT-3’) and shADGRG6-s2 (targeting 5’-CCAAGCAATAATGAATCGTAT-3’), were synthesized by Genechem (Shanghai, China) and incorporated into the GV493 construct individually (Genechem). Subsequently, the constructed shRNA, accompanied by lentivirus packaging constructs from Genechem, underwent co-transfection into HEK-293 cells. This co-transfection process facilitated the production of shRNA lentivirus, enriched (at MOI = 20), and introduced into cultured cells seeded at 60–70% confluence in six-well plates. Scramble control shRNA lentivirus (“shC”) was introduced into control cells. Stable cells were selected using puromycin (for 72 h) and ADGRG6 knockdown efficiency was assessed by qRT-PCR and western blotting.

### Gene overexpression

The lentiviral vector containing the ADGRG6 cDNA (NM_020455) was procured from Genechem (Shanghai, China). The coding sequence of ADGRG6 was cloned into the GV492 lentiviral vector provided by Genechem. The methodologies employed for lentiviral packaging, infection, and establishment of stable cell lines were consistent with those utilized for the ADGRG6 silencing construct.

### ADGRG6 knockout

The sequence of the single-guide RNA (sgRNA) targeting human ADGRG6 (target DNA sequence: CTGAATGATATAACCGGTGG, PAM sequence: GGG) was integrated into the lenti-CRISPR/Cas9-KO-puro vector GV708 (Genechem). The CRISPR/Cas9-ADGRG6-KO construct was transfected into PC cells, followed by selection in puromycin-containing medium to isolate cells with stable integration. Subsequently, a population of cells consisting of a single stable ADGRG6-knockout (KO) clone was established. A lenti-CRISPR/Cas9-KO-puro vector containing a non-targeting sgRNA (“Cas9-C”) represented the control.

### Immunohistochemistry (IHC)

Sections of paraffin-embedded tissue were baked at 60 °C for 1 h, deparaffinized, rehydrated, and subjected to antigen retrieval in citric acid buffer for 10 min at 95 °C. Subsequently, the sections were exposed to 3% H_2_O_2_ to neutralize endogenous peroxidase activity and incubated overnight with the primary antibody at 4 °C. On the subsequent day, after allowing the sections to reach room temperature and washing, they were probed with biotin-tagged sheep anti-rabbit IgG for 1 h, and then treated with HRP-conjugated streptavidin. Diaminobenzidine (DAB) was used to visualize immunoreactivity, and IHC images were captured for further analysis. The immunoreactivity score (IRS = intensity × proportion) system was employed to evaluate the IHC results. Assessment was performed independently by two pathologists blinded to patient diagnoses and clinical outcomes. The IRS represents a composite index derived from the product of staining intensity and the proportion of positively stained tumor cells. Samples were categorized based on IRS, staining intensity (0–3) and percentage of positive tumor cells (0–4) were multiplied to yield an IRS (0–12). For statistical analysis, a cutoff value of IRS = 6 was used to stratify patients into high-expression (IRS ≥ 6) and low-expression (IRS < 6) groups.

### Akt1 mutation

Cultured PC cells were inoculated with a lentivirus comprising a constitutively active S473D mutant of Akt1 (caAkt1) [32, 33], along with a control lentivirus containing an empty vector. Cell lines stably expressing caAkt1 were generated by selecting for puromycin resistance.

### Xenograft model

All in vivo methods were approved by the Ethical Board of the Affiliated Kunshan Hospital Affiliated with Jiangsu University and the Institutional Animal Care and Use Committee (IACUC) (Number: KSPH-IACUC-XM-20250017). Female nude mice, aged six weeks, were provided by the Animal Center of Jiangsu University and kept in a standard environment. The priPC-1 cells (6 × 10^6^ cells/mouse) were injected subcutaneously into the flank region of the upper limbs. Tumor volumes and body weights were evaluated at five-day intervals using digital calipers. Stable priPC-1 cells overexpressing the target gene and control cells harboring an empty vector were established via lentiviral transduction and subsequent puromycin selection. Female BALB/c nude mice (6 weeks old) were housed under specific pathogen-free conditions. Mice were subcutaneously inoculated with 6×10^6^ cells/mouse into the right flank. Upon the formation of palpable tumors, mice bearing the overexpression vector were randomized into two subgroups (*n* = 6 per group). These groups received intraperitoneal injections of either the PI3K inhibitor LY294002 (HY-10108, MedChemExpress) (50 mg/kg) or an equivalent volume of DMSO vehicle every 48 hours. Tumor dimensions and body weights were measured every three days, respectively. Tumor volume was calculated as V = (Length×Width²)/2. At the experimental endpoint, tumors were excised, weighed, and processed for further analysis. A subset of xenograft tissues was snap-frozen for protein extraction and Western blotting to verify target gene expression and confirm inhibition of the PI3K/Akt pathway. Immunohistochemistry (IHC) assays were performed according to previously established protocols [34].

### Metastasis model

To establish the experimental metastasis model, the priPC-1 cells (10^6^ cells/mouse) was injected via the tail vein, allowing systemic dissemination and colonization of target organs, primarily the lungs. Mice were monitored regularly, and humane endpoints were predefined according to the approved protocol. At the study endpoint, mice were euthanized, and metastatic burden was assessed through macroscopic examination. Excised lung tissues were processed for hematoxylin and eosin (H&E) staining to histologically confirm the presence and extent of metastatic lesions.

### Statistical analysis

In vitro analyses were carried out in biological triplicate. Data with normal distributions are shown as the mean ± SD. GraphPad Prism version 10 for Mac was utilized for all the statistical assessments. For analyses involving ADGRG6 expression (excluding immunohistochemistry), the median value served as the cutoff to dichotomize samples into high and low expression groups; this threshold was applied consistently across relevant datasets. Two-group comparisons were performed with t-tests, while one-way ANOVA with the Scheffe and Tukey tests was applied for multiple-group analyses. Disease recurrence rates were compared using the Chi-square, with *p* < *0.05* representing significance.

## Results

### ADGRG6 overexpression and prognostic significance identified via public bioinformatics databases in human PC

**Fig. 1:**
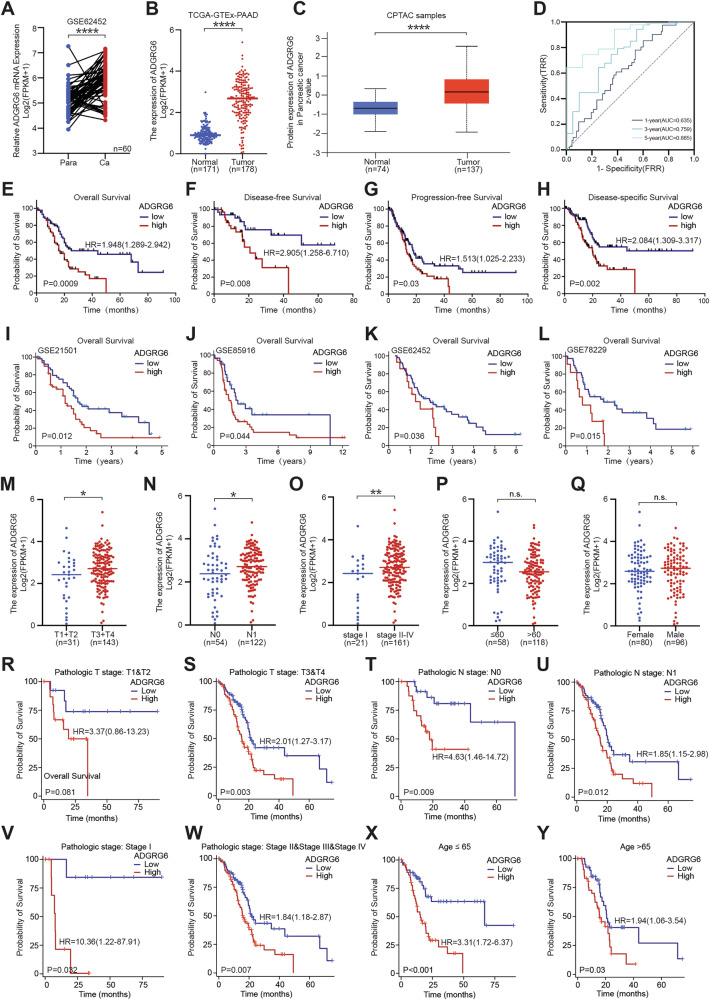
ADGRG6 is overexpressed in human PC. The GEO dataset (GSE62452) revealed variations in ADGRG6 transcript numbers between 60 PDAC tissues and their corresponding adjacent para-cancer tissues (**A**). The TCGA-GTEx-PAAD group demonstrated the relative expression levels of ADGRG6 mRNA transcripts in 171 normal pancreatic tissue (“Normal”) and 178 PC tissue (“Tumor”) samples (**B**). CPTAC dataset (https://ualcan.path.uab.edu) shows ADGRG6 protein expression in 137 PC tumor tissues and 74 normal pancreatic tissues (**C**). The Receiver Operating Characteristic (ROC) curves were presented to evaluate the prognostic value of ADGRG6 overexpression concerning the OS probability in PC patients (**D**). Kaplan Meier Survival analyses of OS (**E**), disease-specific survival (**F**), progression-free survival (**G**), and disease-specific survival (**H**) were carried out based on ADGRG6 mRNA levels in the TCGA-PAAD dataset. Kaplan Meier Survival analyses of OS were also performed based on ADGRG6 expression in GSE21501 (**I**), GSE85916 (**J**), GSE62452 (**K**), and GSE78229 datasets (**L**). The relationship between various clinical features and ADGRG6 expression is presented (**M–Q**). According to the ADGRG6 levels in the TCGA-PAAD dataset, subgroup analysis demonstrated its prognostic value in patients with various clinical subtypes of PC (**R–Y**). PC, pancreatic cancer; PDAC, pancreatic ductal adenocarcinoma; OS, overall survival; HR, hazard rate; ROC, receiver operating characteristic; FPKM, fragments per kilobase of transcript; TPR, true positive rate; AUC, area under the curve; FPR, false positive rate. *p < *0.05*. n.s., non-statistical difference (*p* > *0.05*).

Bioinformatics analyses were first employed to elucidate ADGRG6 expression levels in PC. Examination of *ADGRG6* mRNA expression from 60 paired PC and adjoining normal tissues from the GEO database (GSE62452) revealed significantly elevated ADGRG6 expression in cancerous tissues (Fig. 1A). Similarly, analysis of TCGA and GTEx data confirmed a marked increase in *ADGRG6* mRNA levels in PC tissues (“Tumor”) relative to normal pancreatic tissues (“Normal”) (Fig. 1B). We further investigated the expression of ADGRG6 protein in PC and normal pancreatic tissues using the CPTAC database, which included 137 PC samples (“Tumor”) and 74 normal pancreatic samples (“Normal”). As shown, ADGRG6 protein levels were substantially upregulated in PC tissues relative to normal tissues (Fig. 1C). Subsequently, we evaluated the clinical and prognostic data from the TCGA database. The Receiver Operating Characteristic (ROC) curve assessment revealed that ADGRG6 overexpression could be employed as a predictive marker for 1-, 3-, and 5-year survival rates in PC patients, with areas under the curve (AUC) values of 0.635, 0.759, and 0.885, respectively (Fig. 1D). Survival analysis further revealed that elevated ADGRG6 expression was significantly linked with poorer overall survival (OS; hazard ratio, HR: 1.948, *P* = 0.0009) (Fig. 1E), disease-free survival (DFS; HR: 2.905, *P* = 0.008) (Fig. 1F), progression-free survival (PFS; HR: 1.513, *P* = 0.03) (Fig. 1G), and disease-specific survival (DSS; HR: 2.905, *P* = 0.008) (Fig. 1E) (Fig. 1H). Additionally, PC patients with elevated *ADGRG6* mRNA levels had poorer prognosis across multiple datasets, including GSE21501 (Fig. 1I), GSE85916 (Fig. 1J), GSE62452 (Fig. 1K), and GSE78229 datasets (Fig. 1L). Furthermore, higher ADGRG6 mRNA expression was also associated with advanced T, N, and clinical stages, but was independent of age and gender at the time of initial diagnosis (Fig. 1M–Q). Consistent findings across clinical subgroups demonstrated that higher ADGRG6 mRNA levels were linked to poorer prognostic outcomes (Fig. 1R–Y).

### Single-cell RNA sequencing (scRNA-seq) data reveals upregulation of ADGRG6 in epithelial and endothelial cell populations of PC mediating critical intercellular communication within the tumor microenvironment

**Fig. 2:**
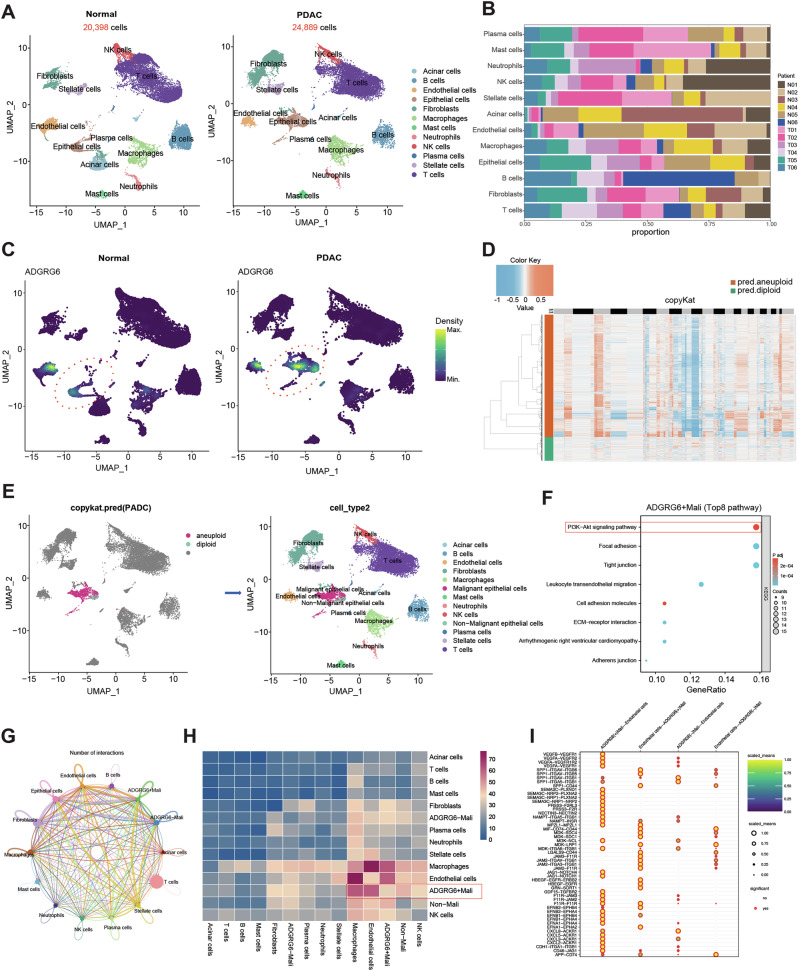
Single-cell RNA sequencing (scRNA-seq) data reveals upregulation of ADGRG6 in epithelial and endothelial cell populations of PC mediating critical intercellular communication within the tumor microenvironment. The single-cell dataset from the GEO database GSE212966 was analyzed. Distribution of 12 cell subsets after cell annotation (**A**). Proportion of each cell type in the TME of all samples (**B**). Characteristic distribution of marker genes (**C**). Classification of epithelial cells into malignant and non-malignant groups using the CopyKAT method (**D**) and visualized by UMAP (**E**). KEGG enrichment analyses reveal the top 8 pathways for ADGRG6-positive epithelial (ADGRG6+epi) cells group (**F**). Integrated map (**G**) and heatmap (**H**) of the overall communication intensity of each cell. Interaction between ADGRG6+epi and ADGRG6-negative epithelial (ADGRG6-epi) and endothelial cells (**I**).

Single-cell transcriptomic analysis based on dataset GSE212966, containing paired tumor and adjacent normal pancreatic tissue samples from six patients, was conducted for further comprehensive analysis of ADGRG6 expression in PC. Through rigorous quality filtering, 45,287 single-cell transcriptomes were retained, comprising 20,398 cells from non-malignant adjacent tissues and 24,889 cells from PADC samples. These cells were categorized into 11 types: Mast, Acinar, B, T, Stellate, NK, Endothelial, Epithelial, and Plasma cells, as well as Fibroblasts, Neutrophils, and Macrophages (Fig. 2A, B). Our single-cell analysis demonstrated significantly elevated ADGRG6 expression levels within the epithelial cells of PADC samples compared to their non-malignant counterparts (Fig. 2C). To further delineate malignant epithelial populations, we utilized the CopyKAT algorithm to infer copy number variations (CNVs) at a single-cell resolution. This approach enabled accurate discrimination between malignant (aneuploid) and non-malignant (diploid) epithelial cells, as visualized through UMAP dimensionality reduction (Fig. 2D, E). Notably, pathway enrichment analysis revealed marked activation of the PI3K/AKT signaling, specifically within the ADGRG6-positive epithelial cell subsets (Fig. 2F). Further exploration of the distribution of ADGRG6-positive (ADGRG6+epi) and ADGRG6-negative (ADGRG6-epi) epithelial cell populations identified distinct cellular subsets characterized by divergent intercellular communication profiles. Leveraging CellphoneDB for ligand-receptor pair analysis in conjunction with scRNA-seq data, we observed a significantly greater abundance of predicted ligand-receptor interactions between ADGRG6+epi cells and endothelial cells, relative to interactions involving ADGRG6-epi cells (Fig. 2G, H). Among these interactions, prominent signaling pathways included VEGFB-VEGFR1 and VEGFA-VEGFR2 axes, highlighting a crucial role for ADGRG6-mediated epithelial-endothelial communication within the tumor microenvironment (Fig. 2I). Bioinformatic analysis consistently indicated that ADGRG6 was highly expressed in PC, particularly within the epithelial and endothelial cell compartments. Elevated ADGRG6 expression correlates with adverse patient outcomes, underscoring its dual role as both a biomarker and a potential therapeutic target for disrupting critical intercellular communication pathways in PC progression.

### Clinical and molecular validation of ADGRG6 overexpression in in-house PC tissues and cells

**Fig. 3:**
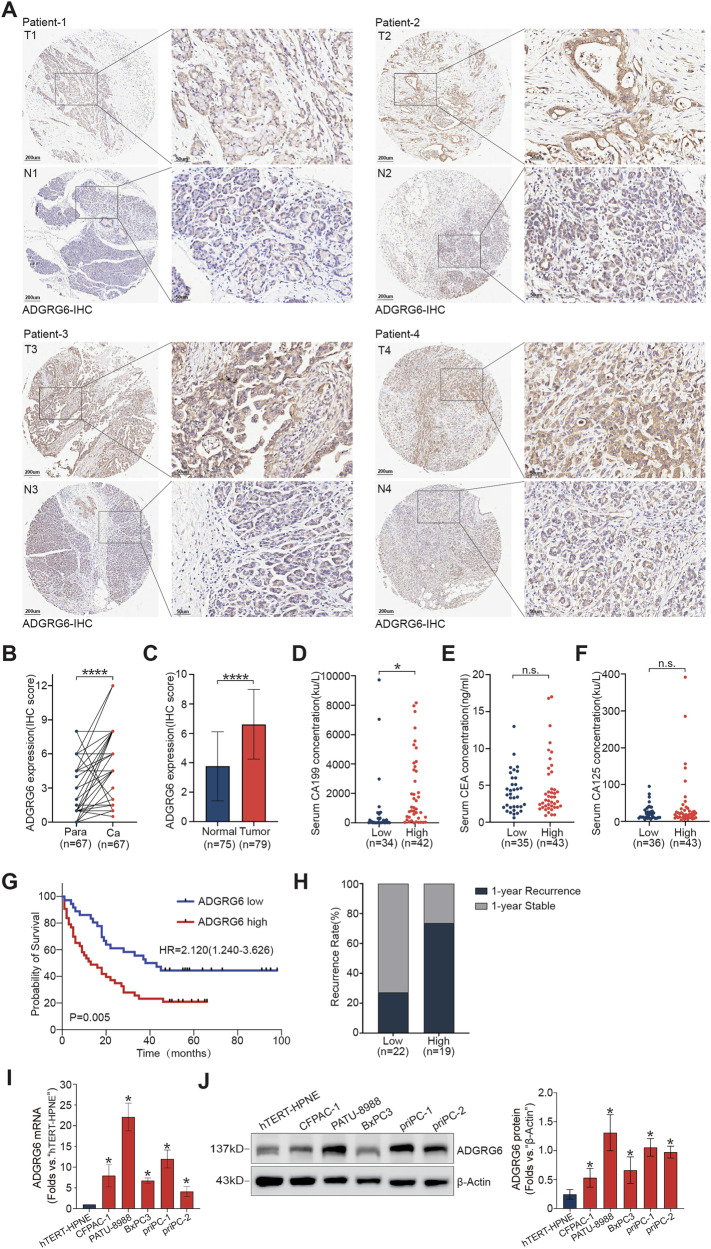
ADGRG6 is overexpressed in PC cell lines and tissues. The tissue microarray indicates ADGRG6 protein levels in PC and paired normal tissues from 4 PC patients (Patient-1, -2, -3, and -4) (**A**). Immunohistochemical scores for ADGRG6 expression were analyzed in 67 paired (“Para”/“Ca”) (**B**) and unpaired (“Normal”, *n* = 75/ “Tumor”, *n* = 79) (**C**) PC and adjacent tissues. Differences in serum levels of CA199, CEA, and CA125 between the ADGRG6 “low” and “high” groups are shown (**D–F**). Survival analysis comparing the survival outcomes between the ADGRG6 “low” and “high” groups is presented in (**G**). Postoperative one-year recurrence data for 41 PC patients, stratified by ADGRG6 levels, is summarized in (**H**). ADGRG6 mRNA (**I**) and protein (**J**) expression were quantified in normal human pancreatic epithelial cells (“hTERT-HPNE”) and PC cells. Data are expressed as mean ± standard deviation (SD). *p < *0.05*. n.s., non-statistical difference (*p* > *0.05*). The number of participants in each subgroup was annotated. The scale bar is marked at the bottom-left corner. PC: pancreatic cancer.

To assess ADGRG6 expression in PC, a tissue microarray consisting of 90 PC samples and paired adjacent non-cancerous tissues was utilized. A significant elevation in ADGRG6 protein levels was observed in PC tissues compared to adjacent non-cancerous tissues, as demonstrated by representative IHC images from four PC patients (Patient-1, -2, -3, and -4) (Fig. 3A). Further evaluation of 67 paired samples of PC (“Ca”) and adjacent tissues (“Para”) revealed significantly higher ADGRG6 protein expression in cancer tissues (Fig. 3B). A similar result was observed when comparing 75 non-paired adjacent tissues (“Normal”) and 79 PC tissues (“Tumor”) (Fig. 3C). Moreover, patients with high ADGRG6 expression exhibited elevated serum levels of CA199, although no marked differences were observed in CEA or CA125 (Fig. 3D–F). High ADGRG6 levels were associated with poorer OS (HR, 2.120; *P* = 0.005) (Fig. 3G). Further statistical analysis of 41 PC patients with follow-up records showed that the recurrence rate within one-year post-surgery was markedly increased in those with high ADGRG6 levels relative to those with low levels (73.68% *vs*. 27.27%, *P* < 0.001) (Fig. 3H).

To further evaluate ADGRG6 expression at the molecular level, qRT-PCR was performed to assess *ADGRG6* mRNA levels in 5 different PC cell lineages, including immortalized CFPAC-1, PATU-8988, and BxPC-3 cell lines, 2 patient-derived primary PC cells (priPC-1 and priPC-2), and the human normal pancreatic ductal cells (hTERT-HPNE). The results showed markedly elevated *ADGRG6* mRNA (Fig. 3I) and protein (Fig. 3J) expression in the different PC cells compared to that in the hTERT-HPNE cells. Taken together, these findings provide strong evidence of ADGRG6 overexpression in human PC tissues, as well as in a variety of PC cell lines, supporting its potential role in PC pathogenesis.

### ADGRG6 silencing *via* shRNA inhibits PC cell proliferation, growth, and motility

**Fig. 4:**
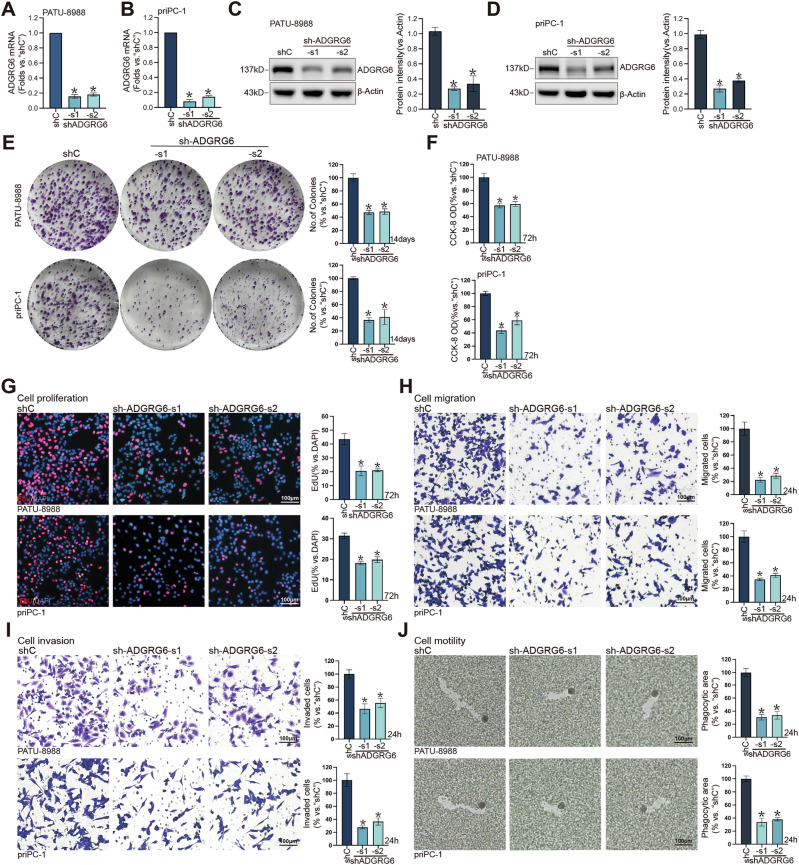
ADGRG6 silencing *via* shRNA inhibits PC cell proliferation, growth, and motility. PATU-8988 and priPC-1 were stably transfected with lentivirus carrying ADGRG6 shRNA (“sh-ADGRG6-s1/s2”, comprising 2 specific sequences) or the scramble control shRNA (“shC”), were selected by puromycin and cultured, and ADGRG6 mRNA levels (**A**, **B**) and protein expression (**C**, **D**) were evaluated. Cells were further propagated for applied periods, colony formation (**E**), viability (**F**), proliferation (**G**), cell migration (**H**), cell invasion (**I**), and cell motility (**J**) analyses *via* the assays listed in the text and the acquired results were analyzed. *p < *0.05 vs*. “shC” cohort. The assays presented in this figure were performed in triplicate (*n* = 3, biological replicates) and yielded consistent results. Scale bar = 100 μm.

As the bioinformatics analyses indicated high levels of ADGRG6 in tumor cells in association with various clinical features and poor prognosis in PC, we sought to examine the functions of ADGRG6 in PC cells and relationships with tumor progression. Lentiviral particles encoding ADGRG6 shRNAs (“ADGRG6-sh-s1/-s2,” with distinct targeting sequences) and a scramble control shRNA (“shC”) were used to infect PATU-8988 and priPC-1 cells, both of which exhibited high ADGRG6 expression among the five PC cell lines examined (Fig. 3J). After established by puromycin selection, stable cell lines were confirmed marked reductions in ADGRG6 mRNA and protein(Fig. 4A–D). ADGRG6 silencing via shRNA significantly impaired colony formation (Fig. 4E) and reduced cell viability, shown by CCK-8 assays (Fig. 4F), in both PATU-8988 and priPC-1 cells. Moreover, a reduction in the number of EdU-positive nuclei, indicative of decreased proliferation, was observed in both cell lines (Fig. 4G). In addition to the effect on proliferation, ADGRG6 silencing significantly compromised the migratory and invasive capacities of both cell lines, as demonstrated by “Transwell” and “Matrigel Transwell assays” (Fig. 4H, I). The phagokinetic track motility assay confirmed that ADGRG6 depletion reduced the motility of PATU-8988 and priPC-1 cells (Fig. 4J). Overall, these results suggest that ADGRG6 is crucially involved in promoting PC cell migration, proliferation, and invasion.

### ADGRG6 knockout significantly suppresses PC cell activity in vitro

**Fig. 5:**
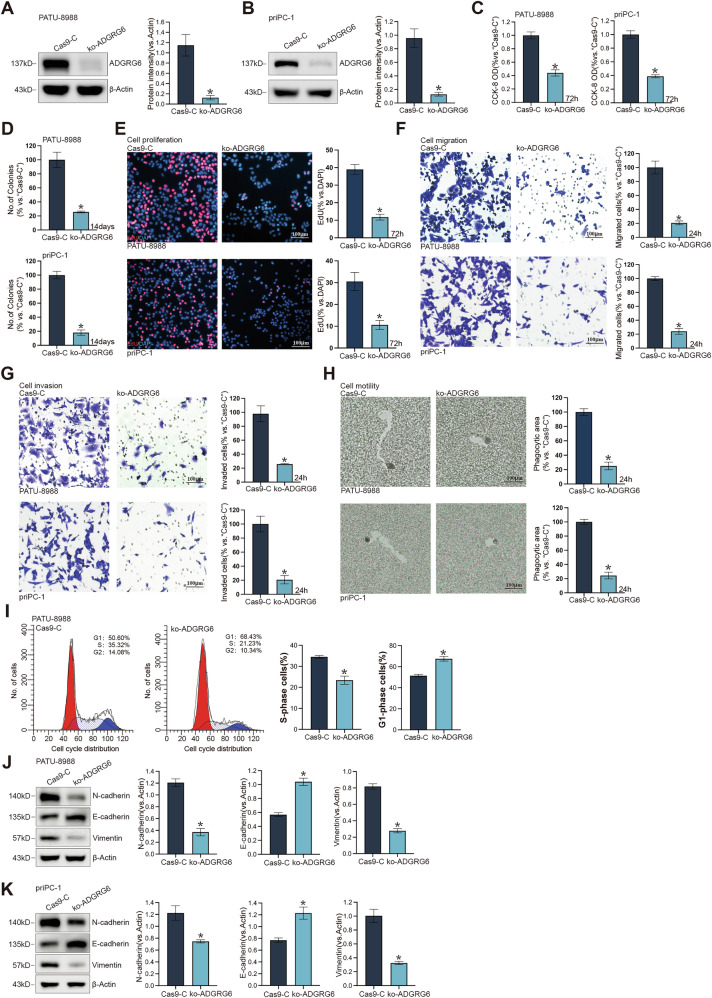
ADGRG6 knockout significantly suppresses PC cell activity in vitro. PATU-8988 and priPC-1 cell lines were transfected with lentiviral CRISPR/Cas9 constructs specifically designed to target ADGRG6 for knockout (“KO-ADGRG6”), or the CRISPR/Cas9 control empty vector (“Cas9-C”). After flow cytometry sorting and puromycin selection, single-cell cloning was performed to establish a stable cell line. Expression of ADGRG6 protein (**A, B**) was tested. Then stable expressed cell lines were further cultured for the next experiment, including cell viability (**C**), colony formation (**D**), proliferation (**E**), migration (**F**), invasion (**G**), and motility (**H**), as well as cell cycle progression (**I**) analyses *via* the assays listed in the text and the acquired results were quantified. Simultaneously, several EMT-related markers were assessed by Western blot analysis (**J, K**). *p < *0.05 vs*. “Cas9-C” cohort. The assays presented in the images were performed in triplicate (*n* = 3 biological repeats) and yielded consistent results. Scale bar = 100 μm.

To further explore ADGRG6’s functional role in PC, we employed CRISPR/Cas9 gene editing to construct stable ADGRG6-knockouts (“koADGRG6”) in PATU-8988 and priPC-1 cells. These cell lines were established through single-clone selection following flow cytometry sorting. Western blotting indicated markedly reduced ADGRG6 levels in both knockout cell lines compared to those harboring the CRISPR/Cas9 control construct (“Cas9-C”) (Fig. 5A, B). Upon ADGRG6 knockout, marked suppression of several key cellular activities was observed. CCK-8 analysis showed a marked reduction in cell viability (Fig. 5C), and the colony formation assay demonstrated a reduction in clonogenic potential (Fig. 5D). Besides, the proportion of EdU-positive nuclei was substantially lower in ADGRG6 knockout cells (Fig. 5E). The migration and invasion capacities were also markedly impaired in the ADGRG6 knockout cells (Fig. 5F, G). The motility of both PATU-8988 and priPC-1 cells were also significantly impaired in the ADGRG6 knockout group (Fig. 5H).

Furthermore, Cell cycle analysis by PI-FACS demonstrated elevated levels of G1 phase cells and a corresponding reduction in S phase cells in the ADGRG6 knockout group, indicating G1-S phase arrest (Fig. 5I). To explore the potential impact of ADGRG6 on epithelial-to-mesenchymal transition (EMT), we analyzed the expression of EMT markers by Western blotting. The results showed a significant upregulation of E-cadherin, together with reductions in N-Cadherin and Vimentin expression (Fig. 5J, K). Together, these findings further demonstrate that ADGRG6 knockout significantly impairs key aspects of PC cell behavior, including proliferation, migration, invasion, and regulation of EMT, highlighting its critical role in PC progression.

### Exogenous overexpression of ADGRG6 exerts pro-cancerous activity in PC cells

**Fig. 6:**
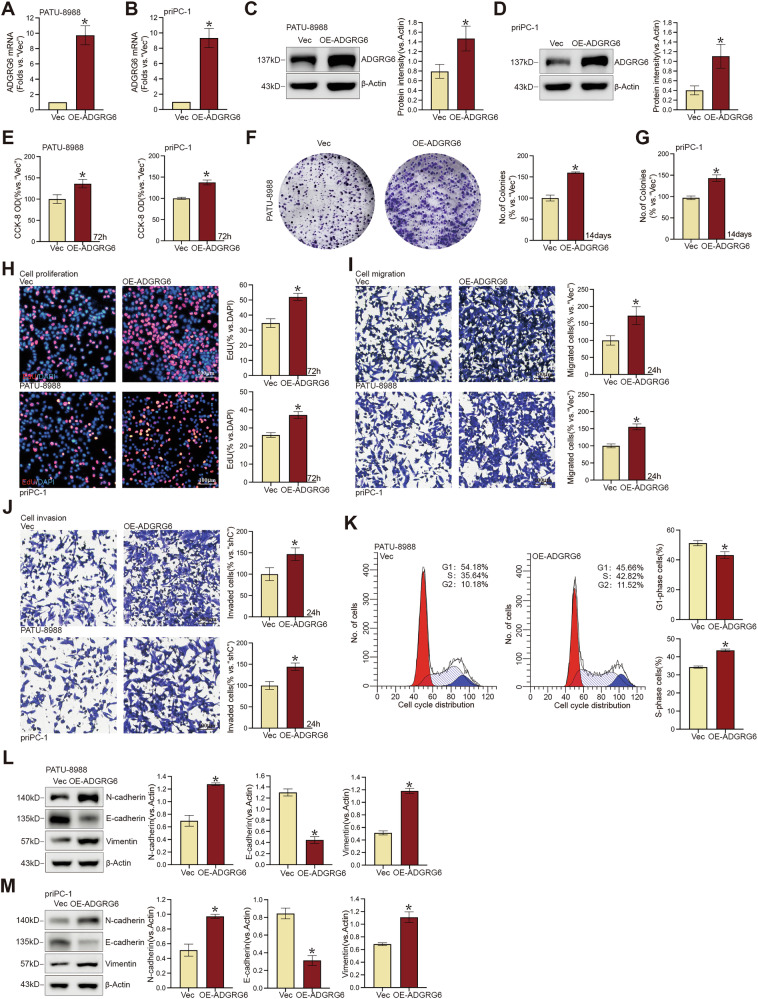
Exogenous overexpression of ADGRG6 exerts pro-cancerous effects in PC cells. PATU-8988 and priPC-1 cells were engineered to overexpress ADGRG6 using the corresponding control vector (“Vec”) or the lentiviral ADGRG6 overexpression construct (“OE- ADGRG6”). The mRNA levels (**A**, **B**) and protein expression (**C**, **D**) of the specified genes are presented. Cells were further propagated for optimized time for cell viability (**E**), colony formation (**F**, **G**), proliferation (**H**), migration (**I**), invasion (**J**), and cell cycle progression (**K**) analyses. *p < *0.05 vs*. “Vec” cohort. The expression of EMT-related markers was assessed by Western blot analysis (**L**, **M**). The assays presented in the presented image were performed in triplicate (*n* = 3, biological repeats) and yielded consistent results. Scale bar = 100 μm.

A lentiviral construct encoding ADGRG6 cDNA (“OE-ADGRG6”) was transduced into PATU-8988 and priPC-1 cells. Stable cell lines were selected with puromycin, and ADGRG6 mRNA and protein levels were assessed. qRT-PCR (Fig. 6A, B) and Western blotting (Fig. 6C, D) showed marked upregulation of ADGRG6 in the overexpressing cells relative to the controls with the empty vector (“Vec”). Functional assays demonstrated that ADGRG6 overexpression significantly promoted the CCK-8 cell viability (Fig. 6E) and colony formation (Fig. 6F, G) in PC cells. The Edu incorporation assay also revealed a substantial increase in Edu-positive nuclei, further supporting the pro-proliferative effect of ADGRG6 overexpression (Fig. 6H). Furthermore, “Transwell” and “Matrigel Transwell” analyses demonstrated that ADGRG6 overexpression promoted both the migratory and invasive capabilities of the PC cells (Fig. 6I-J).

Additionally, flow cytometric analysis of the cell cycle showed a marked rise in the proportion of cells in the S phase (Fig. 6K), indicating accelerated cell cycle progression. Moreover, along with ADGRG6 overexpression, E-cadherin levels was substantially decreased while N-cadherin and Vimentin levels were increased (Fig. 6L). These results mutually confirm the pro-tumorigenic role of ADGRG6 in vitro.

### ADGRG6 is essential for activating Akt-mTOR in PC cells

**Fig. 7:**
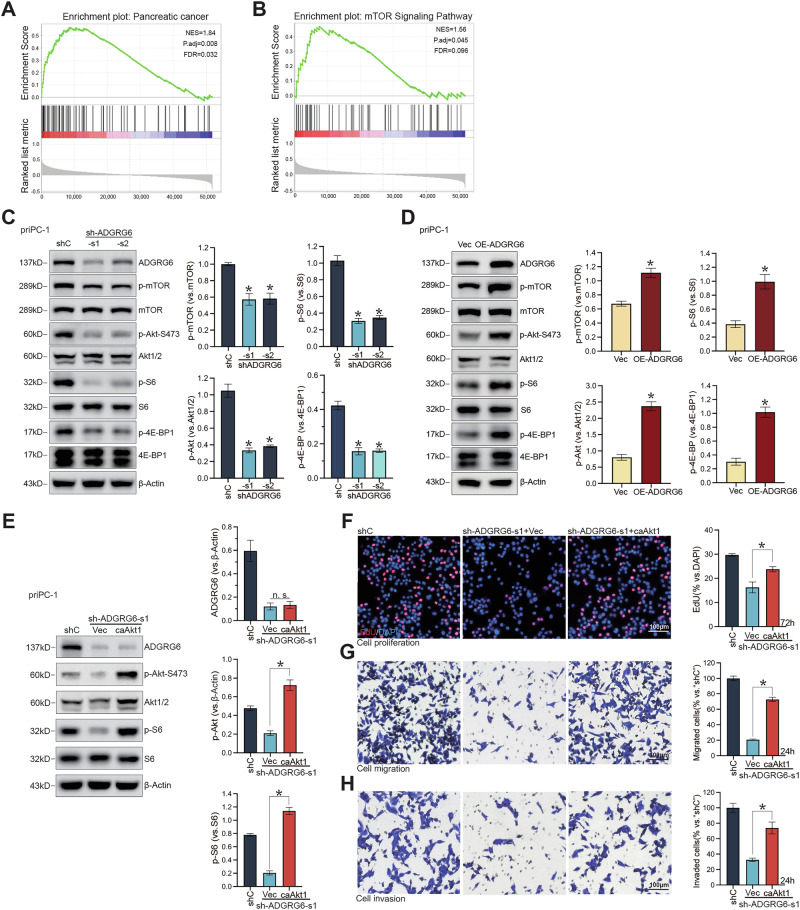
ADGRG6 is essential for activating Akt-mTOR in PC cells. KEGG enrichment and GSEA were used to assess the functions of ADGRG6-associated differentially expressed genes (DEGs) using data from the TCGA-PAAD dataset (**A**, **B**). PriPC-1 cells were engineered to express ADGRG6 shRNA (“sh-ADGRG6-s1/s2”), scramble control shRNA (“shC”), OE-ADGRG6 (“OE-ADGRG6”) and the lentiviral vector (“Vec”). The expression of critical proteins within the Akt-mTORC1 signaling pathway were examined (**C**, **D**). priPC-1 cells transduced with “sh-ADGRG6-s1” were generated to either express a control vector (“Vec”) or a constitutively active Akt1 mutant (caAkt1, S473D). Then, the levels of targeted proteins were evaluated (**E**). Following this, the cells were propagated for 24 to 72 h for cell proliferation (**F**), migration (**G**), and invasion (**H**) analyses, and the acquired results were quantitatively analyzed. *p < *0.05 vs*. “shC”/ “Vec” cohort. The scale bar is marked at the bottom right corner.

To elucidate the molecular and cellular mechanisms underlying ADGRG6-driven progression in PC, we conducted Gene Set Enrichment Analysis (GSEA) utilizing transcriptomic data obtained from PC patient samples in The Cancer Genome Atlas (TCGA) database. By characterized by high or low ADGRG6 expression levels, patients were stratified into groups. Our analysis revealed that elevated ADGRG6 expression significantly correlated with activation signatures of multiple oncogenic pathways, prominently including the PC pathway (Fig. 7A) and the mTOR signaling pathway (Fig. 7B). These findings collectively suggest that ADGRG6 may facilitate PC progression, at least partially, by regulating Akt-mTOR signaling. In priPC-1 cells, western blot analysis showed that knockdown of ADGRG6 markedly reduced phosphorylation levels of mTOR, Akt (Ser-473), S6, and 4E-BP1 (Fig. 7C). Conversely, stable ADGRG6 overexpression in priPC-1 cells (OE-ADGRG6) robustly enhanced the phosphorylation of these signaling molecules relative to that in control cells (Fig. 7D).

To further validate whether Akt-mTOR activation is the key mechanism underlying ADGRG6-driven PC progression, a constitutively active Akt1 mutant (caAkt1) was introduced into ADGRG6-silenced priPC-1 cells (“sh-ADGRG6-s1”) through lentiviral transduction. Western blot analysis demonstrated that ectopic expression of caAkt1 effectively restored Akt and S6 phosphorylation in ADGRG6-deficient cells, whereas ADGRG6 protein expression remained unchanged (Fig. 7E). Importantly, caAkt1 expression significantly reversed the inhibitory effects observed upon ADGRG6 knockdown, restoring cellular proliferation, migration, and invasion capabilities(Fig. 7F, G). These results provide strong evidence that supports the crucial position of the activated Akt-mTOR pathway in mediating the oncogenic functions of ADGRG6 during PC proliferation.

### Gαi3 is crucial for mediating ADGRG6-driven activation of Akt-mTOR in PC cells

**Fig. 8:**
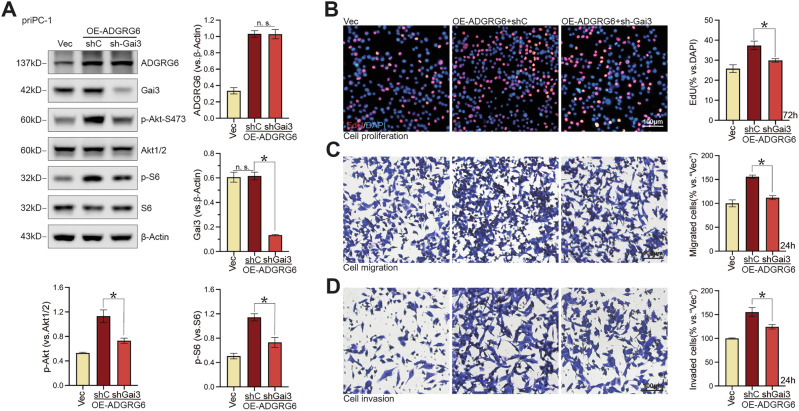
Gαi3 is crucial for mediating ADGRG6-driven activation of Akt-mTOR in PC cells. priPC-1 cells overexpressing ADGRG6 (OE-ADGRG6) were subsequently subjected to stable transduction with either the scrambled control shRNA (“shC”) or lentiviral Gαi3 shRNA (“sh-Gαi3”). The expression of specific proteins was then assessed (**A**). Subsequently, these cells were grown for designated periods, and proliferation, migration, and invasion were assessed using EdU assays (**B**), “Transwell” (**C**), and “Matrigel Transwell” assays (**D**), respectively. The scale bar is marked in the bottom right corner.

Next, we sought to elucidate the molecular mechanism underlying ADGRG6-mediated activation of the Akt-mTOR signaling pathway in PC cells. Our previous studies identified the Gαi subunit isoform Gαi3 as being significantly upregulated in PC, where it functioned as a critical activator of the Akt-mTOR pathway, thereby promoting PC cell proliferation. Building upon these prior observations, we specifically investigated whether Gαi3 is required for the ADGRG6-driven activation of Akt-mTOR signaling in PC cells. To test this hypothesis, we employed priPC-1 cells stably overexpressing ADGRG6 (OE-ADGRG6) and performed targeted knockdown of Gαi3 using a lentivirus-packaged Gαi3 shRNA (sh-Gai3) [32, 35]. Our results demonstrated that silencing Gαi3 substantially inhibited the Akt-mTOR signaling cascade, as evidenced by a pronounced decrease in phosphorylated Akt and S6 levels (Fig. 8A). In addition, suppression of Gαi3 reversed the enhanced malignant phenotypes induced by ADGRG6 overexpression, significantly attenuating PC cell proliferation, migration, and invasion (Fig. 8B, C). Collectively, these findings offer compelling support for the pivotal role of Gαi3 in mediating ADGRG6-driven activation of the Akt-mTOR signaling axis.

### ADGRG6 knockdown suppresses in vivo priPC-1 xenograft tumor growth and metastasis

**Fig. 9:**
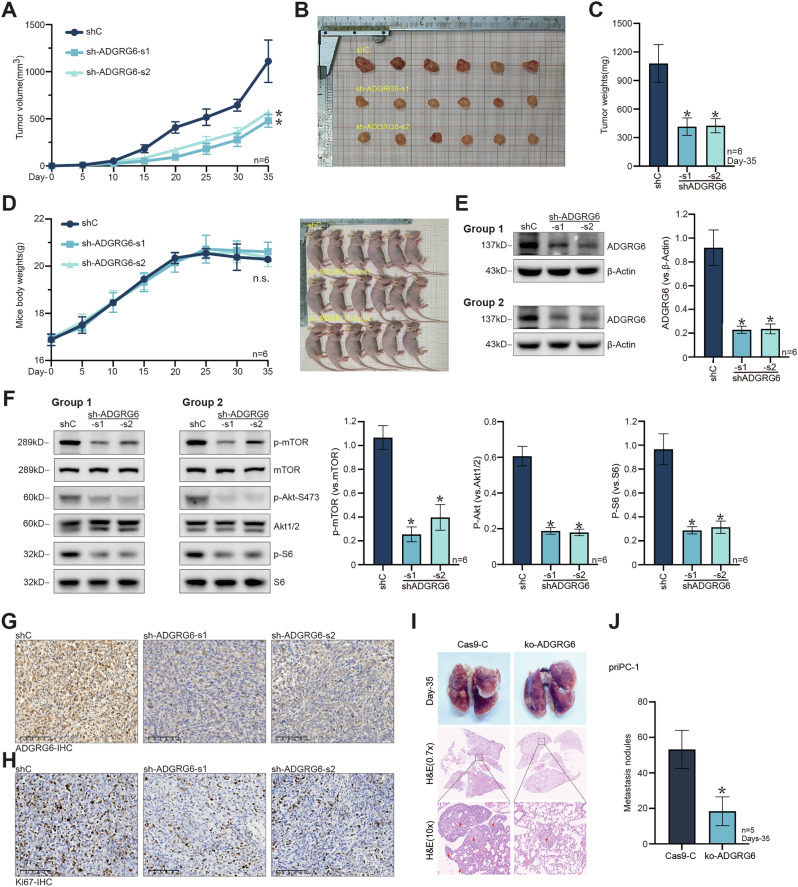
ADGRG6 knockdown suppresses priPC-1 xenograft tumor growth in vivo. Female BALB/c nude mice bearing PriPC-1 xenografts were generated *via* subcutaneous administration of priPC-1 cells expressing either scrambled control shRNA (“shC”) or ADGRG6 shRNA (“sh-ADGRG6-s1” and “sh-ADGRG6-s2”), respectively. Every five days, tumor volumes (**A**) and mice body weights (**D**) were measured. After 35 days, all tumors were dissected (**B**) and weighed (**C**). ADGRG6 protein levels and the listed proteins in tumor tissue lysates were evaluated by Western blot analysis (**E**, **F**), and the results were quantified. Furthermore, xenograft samples were also analyzed by immunohistochemistry to elucidate the levels of ADGRG6 (**G**) and Ki-67 (**H**). Representative images showing the gross morphology of lung surface metastases, along with H&E-stained sections of pulmonary tissue samples (**I**). Quantification of pulmonary metastatic nodules (**J**) observed in H&E-stained sections. Data are presented as mean ± standard deviation (SD), with *p < *0.05 vs*. “shC”/ “Cas9-C” cohort.

To evaluate the functional significance of ADGRG6 in the progression of PC in vivo, a murine xenograft model was established using immunodeficient mice. Explicitly, priPC-1 cells stably expressing ADGRG6-specific short hairpin RNAs (“sh-ADGRG6-s1” or “sh-ADGRG6-s2”) or a scrambled control shRNA (“shC”) were subcutaneously injected into the mice. By measuring tumor volumes every five days, the growth of tumor was monitored from the baseline (“Day 0”) until the experimental endpoint (“Day 35”) (Fig. 9A). At the conclusion of the experiment, tumors in mice injected with sh-ADGRG6-s1 or sh-ADGRG6-s2 priPC-1 cells exhibited significantly reduced volumes and weights compared with those derived from control shC priPC-1 cells (Fig. 9B, C). Importantly, there is no significant differences in body weights among the experimental groups throughout the study period, indicating that ADGRG6 knockdown did not induce systemic toxicity or adversely affect overall animal health (Fig. 9D). Tumor tissues were isolated and analyzed. The western blot analysis indicated a substantial protein expression reduction of ADGRG6 in priPC-1 xenografts expressing sh-ADGRG6 (Fig. 9E). Furthermore, ADGRG6 knockdown led to significant inhibition in the phosphorylation levels of key Akt/mTOR pathway components, including Akt, mTOR, and S6 (Fig. 9F). Immunohistochemical (IHC) staining corroborated these findings, demonstrating markedly reduced ADGRG6 protein levels (Fig. 9G) and decreased Ki67-positive proliferating cells in xenografts derived from ADGRG6-silenced priPC-1 cells compared to controls (Fig. 9H). Additionally, to assess the effect of ADGRG6 on metastatic potential, we established a tail vein injection model using ADGRG6 knockout (“ko-ADGRG6”) and control priPC-1 cells (“Cas9-C”). After 35 days, lung tissues were harvested and analyzed (Fig. 9I). The number and size of lung metastatic nodules were markedly reduced in the ADGRG6 knockout group compared to the control group (Fig. 9J), indicating that ADGRG6 depletion significantly suppresses the metastatic capacity of PC cells in vivo.

### ADGRG6 overexpression promotes in vivo primary PC cell growth *via* AKT/m-TOR

**Fig. 10:**
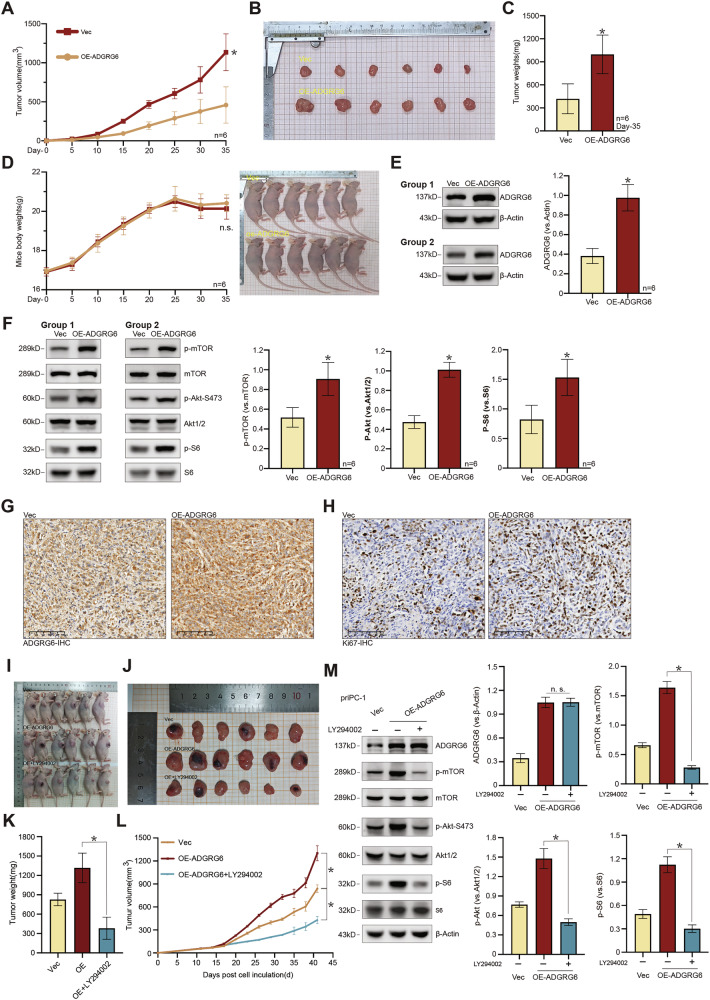
ADGRG6 overexpression promoted *primary PC cell growth* in vivo. Xenografts were established in female BALB/c nude mice by injecting PriPC-1 cells expressing the lentiviral ADGRG6-expressing construct (“OE-ADGRG6 “) or the lentiviral vector (“Vec”). Tumor volumes (**A**) and body weights of the mice (**D**) were monitored every five days. After 35 days, all tumors were dissected (**B**) and weighed (**C**). Protein levels of ADGRG6 and the listed proteins in tumor tissue lysates were analyzed by Western blot (**E**, **F**), and the results were quantified. Furthermore, xenograft sections were analyzed by immunohistochemistry to elucidate levels of ADGRG6 (**G**) and Ki-67 (**H**). Representative images of resected priPC-1 xenografts from mice with ADGRG6 overexpression (“OE-ADGRG6”), OE-ADGRG6 treated with LY294002 (“OE + LY294002”), or vector control (“Vec”) at the endpoint, with quantification of tumor weight and volume (**I–K**). Tumor growth curves of priPC-1 xenografts (**L**) with ADGRG6 overexpression (“OE-ADGRG6”), OE-ADGRG6 treated with LY294002 (“OE + LY294002”), or vector control (“Vec”), measured every three days. Western blot analysis of PI3K-AKT pathway-related protein expression (**M**) in tumor tissue lysates, with densitometric quantification. Animal experiments included five mice per group (*n* = 6). Data are shown as mean ± SD, with *p < *0.05 vs*. “Vec” cohort.

To further elucidate the functional impact of ADGRG6 overexpression on PC progression in vivo, we generated xenograft tumors by subcutaneously injecting priPC-1 cells stably overexpressing ADGRG6 (“ADGRG6-OE”) or control vector (“Vec”) into nude mice. Compared to those in the control group, ADGRG6-OE tumors exhibited significantly increased tumor volumes and weights at the experimental endpoint (Fig. 10A–C). No significant changes in body weights were noted in mice from either group (Fig. 10D). Western blot analysis confirmed elevated expression levels of ADGRG6 protein in ADGRG6-OE xenografts (Fig. 10E), accompanied by enhanced phosphorylation of Akt (Ser-473), mTOR, and S6 (Fig. 10F). IHC analyses further substantiated increased ADGRG6 expression (Fig. 10G) and demonstrated elevated numbers of Ki67-positive proliferating nuclei in ADGRG6-OE tumor sections (Fig. 10H). Taken together, these findings establish that ADGRG6 significantly accelerates PC growth in vivo and is associated with the Akt/mTOR signaling activation, highlighting its potential as a therapeutic target. Importantly, administration of the PI3K inhibitor LY294002 to tumor-bearing mice significantly attenuated the tumor-promoting effects of ADGRG6 overexpression in vivo (Fig. 10I, J). Tumors excised from ADGRG6-overexpressing mice treated with LY294002 were markedly smaller and lighter than those from the ADGRG6-OE group without inhibitor treatment (Fig. 10K, L). Western blot analysis of excised tumors further showed that LY294002 administration led to decreased phosphorylation of Akt, S6, and 4EBP1 in the ADGRG6-overexpressing group (Fig. 10M), indicating effective inhibition of the Akt-mTOR pathway. This result further confirms the functional importance of the Akt-mTOR-S6/4EBP1 pathway in mediating ADGRG6 oncogenicity.

## Discussion

Pancreatic cancer (PC) is a malignant tumor originating from ductal epithelial and acinar cells, and approximately 80% of patients experience disease progression [36]. Despite extensive research efforts over recent decades, the prognosis of PC has shown minimal improvement. Indeed, PC is anticipated to emerge as the second most common cause of cancer-related deaths worldwide by 2040 [37, 38]. A hallmark of PC pathology is its characteristically dense and fibrotic extracellular matrix (ECM) accompanied by low cellular density. This ECM-rich tumor microenvironment significantly limits therapeutic drug penetration and restricts immune cell infiltration, thereby promoting tumor growth, progression, and therapeutic resistance [39]. Given the limited effectiveness and significant toxicity of conventional chemotherapy, molecular-targeted therapies represent a promising alternative for PC treatment [40, 41]. Compared to traditional chemotherapeutic agents, molecularly targeted approaches offer increased specificity, enhanced therapeutic efficacy, and reduced adverse effects [42]. However, the identification of viable molecular targets remains challenging due to the inherent difficulty of directly targeting known PC driver oncogenes and the limited availability of effective alternative therapies [36]. Therefore, the identification of novel therapeutic targets for PC remains an urgent priority.

ADGRG6, an adhesion G protein-coupled receptor, has been recently identified as a promoting factor in the pathogenesis of various cancers [21, 25, 26, 28, 43]. In colorectal cancer, the silencing of ADGRG6 inhibits tumor cell viability, cell cycle progression, and other malignant biological behaviors [28]. Under the activation of specific extracellular hormones, ADGRG6 promotes triple-negative breast cancer cell growth via the Gαi-SRC signaling pathway in vitro and in vivo [43]. Moreover, mutations in ADGRG6 have been identified in bladder cancer [25, 44]. Notably, ADGRG6 also contributes to angiogenesis critically [24].

Our bioinformatics analysis revealed significantly elevated ADGRG6 expression in PC tissues in comparison with that in adjacent non-cancerous tissues, correlating with advanced tumor stages and poorer survival outcomes. Moreover, high ADGRG6 expression correlated positively with elevated serum carbohydrate antigen 19-9 (CA19-9) concentration, a clinically established biomarker routinely employed for PC diagnosis and disease monitoring [45–47]. Although CA19-9 itself has limited characterization of its molecular mechanisms in PC biology, our findings highlight a potentially significant functional relationship between ADGRG6 and CA19-9. This relationship warrants further mechanistic investigations in future studies. Notably, single-cell sequencing data showed that ADGRG6 was primarily concentrated in malignant epithelial cells and is highly likely to be involved in tumor angiogenesis through the VEGF signaling pathway, underscoring its potential acting as a vital diagnostic and prognostic biomarker in PC. Our analysis demonstrated that ADGRG6 as a reliable prognostic marker for PC patient outcomes, and its integration into postoperative monitoring protocols might provide valuable insights into disease progression and recurrence risk.

Here, we confirmed that ADGRG6 is essential for the progression of PC cells, both in vitro and in vivo. Depletion of ADGRG6 via targeted shRNA or CRISPR-Cas9 knockout significantly inhibited cell proliferation, viability, cell cycle progression, migration and invasion. In contrast, ectopic overexpression of ADGRG6 in PC cells further enhanced pro-tumorigenic effects. In vivo, ADGRG6 depletion suppressed the growth of priPC cell-derived subcutaneous xenografts in nude mice.

Akt-mTOR signaling is a well-established regulator of pancreatic cancer (PC) progression [48–50]. Over 90% of pancreatic ductal adenocarcinoma (PDAC) cases harbor mutations in the Kristen rat sarcoma viral oncogene homolog (KRAS) [51], a critical upstream regulator of the Akt-mTOR pathway. KRAS mutations directly activate PI3K/Akt signaling and enhance mTOR activity, promoting tumor growth and survival [50]. In this study, single-cell sequencing analysis of dataset GSE212966 and GSEA enrichment analysis of transcriptomic data from TCGA PC patients revealed that high ADGRG6 expression was enriched in the Akt-mTOR axis, a crucial modulator of cell proliferation, metabolic processes, and survival frequently exhibiting dysregulation in PC [49, 52–55]. ADGRG6 shRNA or KO in primary human PC cells notably decreased Akt and S6 phosphorylation. Similarly, the overexpression of ADGRG6 produced an opposing effect, enhancing Akt and S6 phosphorylation. Additionally, in vivo experiments confirmed that ADGRG6 depletion led to a notable decrease in Akt and S6 phosphorylation within PC xenografts. These findings demonstrate that ADGRG6 is essential for Akt-mTOR signaling pathway activation, strongly suggesting that the facilitation of Akt-mTOR cascade activation represents a critical mechanism underlying ADGRG6-mediated PC cell growth.

Previous studies have demonstrated that ligand-bound ADGRG6 can directly activate Gαi signaling pathways in tumor cells [56]. Additionally, in HER2-positive breast cancer, GPCR-mediated Gαi activation transactivates EGFR and HER2 receptors, subsequently stimulating PI3K-Akt signaling. Our recent findings have identified Gαi3 as a promising candidate for targeted therapy for human PC. Elevated Gαi3 expression has been detected in human PC tissues and cells [35]. Depletion of Gαi3 suppresses Akt-mTOR signaling and inhibits PC cell growth both in vitro and in vivo. Herein, we intended to the character of Gαi3 in ADGRG6-mediated signaling. Specifically, lentiviral-mediated shRNA knockdown of Gαi3 in ADGRG6-overexpressing priPC-1 cells (“OE-ADGRG6” priPC-1 cells) effectively reversed ADGRG6-induced Akt-mTOR activation and alleviated its pro-oncogenic effects (Fig. 8). Collectively, these findings support a crucial mechanistic role for Gαi3 as a downstream mediator of ADGRG6-driven stimulation of the Akt-mTOR signaling cascade in PC.

Despite the strengths of our study, several limitations warrant discussion. First, while we have elucidated the downstream signaling pathways of ADGRG6, the upstream mechanisms governing its activation within the pancreatic cancer microenvironment remain to be fully defined. Recent studies have identified extracellular matrix (ECM) components such as Collagen VI and laminin-211 as potential endogenous ligands of ADGRG6 in various biological contexts [57, 58]. Given that Collagen VI is abundantly deposited in the desmoplastic stroma of PC and functions as an autocrine regulator of tumor growth [59], it is plausible that Collagen VI serves as a key upstream activator of ADGRG6. This hypothesis suggests a potential mechanism by which the fibrotic stroma drives pancreatic cancer progression via the ADGRG6 axis and highlights the ADGRG6–Collagen VI interaction as a potential target for therapeutic disruption.

Additionally, the mechanistic basis for the observed correlation between ADGRG6 expression and serum CA19-9 levels remains to be determined. Currently, there is no direct evidence that ADGRG6 regulates the biosynthesis, glycosylation, or secretion of CA19-9. Therefore, the strong correlation observed in our cohort likely reflects a concomitant upregulation associated with high tumor burden and advanced disease stage, rather than a direct causal relationship.

Finally, targeting GPCR as a potential therapeutic strategy is an area of increasing interest [60]. As a GPCR, ADGRG6 is theoretically amenable to targeting via small molecule inhibitors or monoclonal antibodies. However, the receptor plays critical physiological roles in normal tissues, including myelination, vascular development, and epithelial homeostasis [24, 61–63]. Consequently, systemic inhibition of ADGRG6 could lead to undesirable off-target effects or toxicity. To mitigate such risks, future work should focus on developing highly selective modulators or targeted delivery systems that restrict the effects of ADGRG6 inhibition to tumor tissue. Preclinical studies assessing the tissue distribution, selectivity, and safety profile of candidate inhibitors or antibodies will be a prerequisite to ensuring the clinical feasibility of ADGRG6-targeted therapies.

**Fig. 11:**
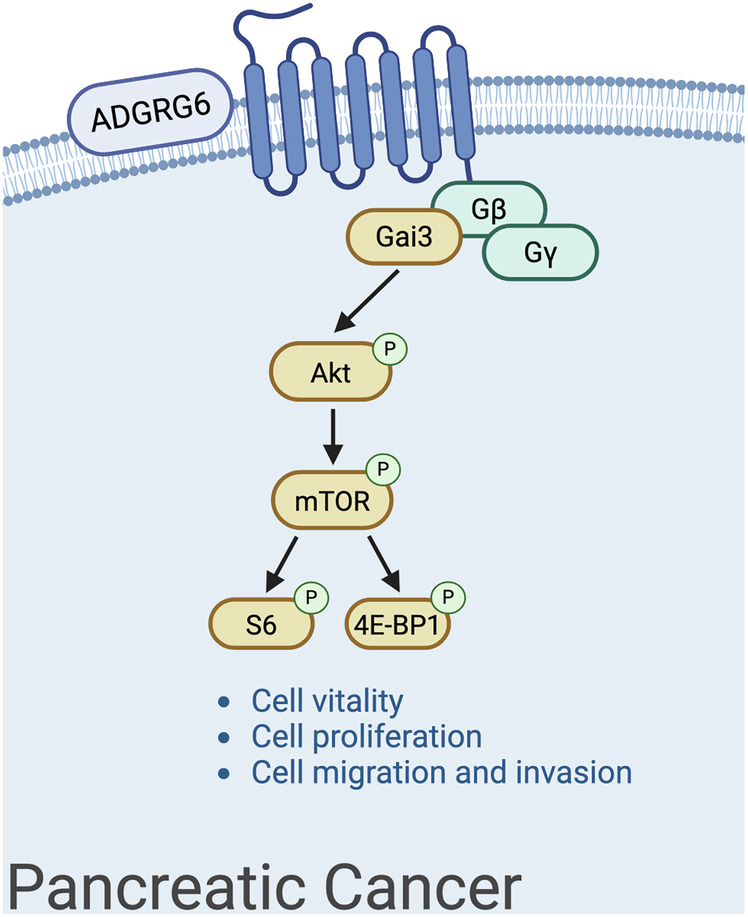
The proposed signaling pathway elucidated the molecular mechanisms involved in this study. ADGRG6 activates the Akt-mTOR signaling pathway mediated by Gαi3, facilitating the proliferation of pancreatic cancer cells both in vitro and in vivo, suggesting its viability as a therapeutic target for pancreatic cancer treatment.

Ultimately, our findings emphasize the crucial function of ADGRG6 in the progression of PC. Elevated ADGRG6 expression is associated with advanced disease stages, increased recurrence rates, and poorer patient prognosis. Mechanistically, by activating the Akt-mTOR signaling pathway through the downstream mediator Gαi3, ADGRG6 promotes PC cell proliferation, migration, and invasion (Fig. 11). Given its significant involvement in tumor progression, ADGRG6 represents a promising therapeutic target in PC. Targeting ADGRG6 might not only inhibit tumor growth but also reduce postoperative recurrence and improve long-term survival outcomes in PC patients.

## Supplementary information

original data of PCR

original data of western bolt

## Data Availability

All data are available upon request.
