# Supplementary material for: Characterization of ADGRG6 as a potential molecular oncotarget of pancreatic cancer
Source: Cell Death Dis. 2026 May 6;17(1):604. doi: 10.1038/s41419-026-08766-2 (PMC13316050; doi:10.1038/s41419-026-08766-2)
Supplement: Supplementary file 1 — original data of PCR [file 41419_2026_8766_MOESM1_ESM.pdf]

ADGRG6 mRNA expression of normal human pancreatic epithelial cells (“hTERT-HPNE”) and PC cells

No.1		Ct	Ct	Ct	**Mean value**			Ct	Ct	Ct	**Mean value**
ADGRG6	**hTERT-HPNE**	25.40	25.57	25.47	25.48	β-Actin	**hTERT-HPNE**	15.28	15.30	15.30	15.29
	**CFPAC-1**	23.35	23.77	23.50	23.54		**CFPAC-1**	16.66	17.00	16.77	16.81
	**PATU-8988**	20.90	20.91	20.85	20.89		**PATU-8988**	15.27	15.52	15.39	15.39
	**BXPC3**	22.69	22.81	22.93	22.81		**BXPC3**	15.37	15.55	15.52	15.48
	**priPC-1**	22.66	22.68	22.69	22.68		**priPC-1**	16.18	16.24	16.58	16.33
	**priPC-2**	24.52	24.42	24.68	24.54		**priPC-2**	16.62	16.30	16.30	16.41

No.2		Ct	Ct	Ct	**Mean value**			Ct	Ct	Ct	**Mean value**
ADGRG6	**hTERT-HPNE**	24.50	24.64	24.40	24.51	β-Actin	**hTERT-HPNE**	14.87	15.22	14.95	15.01
	**CFPAC-1**	23.41	23.25	23.23	23.30		**CFPAC-1**	16.69	16.63	16.63	16.65
	**PATU-8988**	21.95	21.92	21.95	21.94		**PATU-8988**	16.76	16.71	16.70	16.72
	**BXPC3**	23.43	23.61	23.42	23.49		**BXPC3**	16.71	16.83	16.85	16.80
	**priPC-1**	22.81	22.85	23.15	22.94		**priPC-1**	16.84	16.56	16.93	16.78
	**priPC-2**	24.35	24.30	24.25	24.30		**priPC-2**	16.38	16.42	16.26	16.35

No.3		Ct	Ct	Ct	**Mean value**			Ct	Ct	Ct	**Mean value**
ADGRG6	**hTERT-HPNE**	25.71	25.51	25.42	25.55	β-Actin	**hTERT-HPNE**	15.23	15.44	15.32	15.33
	**CFPAC-1**	22.98	23.17	22.82	22.99		**CFPAC-1**	15.14	15.35	15.36	15.28
	**PATU-8988**	21.90	21.58	21.50	21.66		**PATU-8988**	15.85	15.87	15.78	15.83
	**BXPC3**	23.45	23.66	23.62	23.58		**BXPC3**	15.97	15.77	16.12	15.95

	priPC-1	21.33	21.68	21.62	21.54		priPC-1	14.86	14.86	14.81	14.84
	priPC-2	24.93	24.85	24.48	24.75		priPC-2	16.77	17.13	16.97	16.96

Silence ADGRG6 expression

priPC-1

No.1		Ct	Ct	Ct	**Mean value**			Ct	Ct	Ct	**Mean value**
ADGRG6	**shC**	21.57	21.46	21.39	21.48	β-Actin	**shC**	16.48	16.85	16.62	16.65
	**sh-ADGRG6-s'**	25.87	25.53	26.01	25.8		**sh-ADGRG6-s1**	16.67	16.98	16.96	16.87
	**sh-ADGRG6-s:**	24.34	24.29	24.69	24.44		**sh-ADGRG6-s2**	16.8	16.53	16.65	16.66

No.2		Ct	Ct	Ct				Ct	Ct	Ct	
ADGRG6	**shC**	21.94	21.93	21.93	21.93	β-Actin	**shC**	16.15	16.39	16.51	16.35
	**sh-ADGRG6-s'**	25.33	25.41	25.45	25.4		**sh-ADGRG6-s1**	16.74	16.31	16.69	16.58
	**sh-ADGRG6-s:**	24.85	24.81	24.74	24.8		**sh-ADGRG6-s2**	16.24	16.55	16.44	16.41

No.3		Ct	Ct	Ct				Ct	Ct	Ct	
ADGRG6	**shC**	21.9	21.61	21.77	21.76	β-Actin	**shC**	16.61	16.22	16.46	16.43
	**sh-ADGRG6-s'**	25.44	25.45	25.37	25.42		**sh-ADGRG6-s1**	16.23	16.74	16.33	16.43
	**sh-ADGRG6-s:**	24.31	24.59	24.37	24.43		**sh-ADGRG6-s2**	16.25	16.59	16.45	16.43

PATU-8988

No.1		Ct	Ct	Ct	**Mean value**			Ct	Ct	Ct	**Mean value**

ADGRG6	**shC**	21.2	21.49	21.37	21.35	β-Actin	**shC**	16.45	16.08	16.4	16.31
	**sh-ADGRG6-s**	24.68	24.51	24.61	24.6		**sh-ADGRG6-s2**	16.79	16.58	16.7	16.69
	**sh-ADGRG6-s**	23.87	23.64	24.08	23.86		**sh-ADGRG6-s1**	16.26	16.51	16.43	16.4

No.2		Ct	Ct	Ct				Ct	Ct	Ct	
ADGRG6	**shC**	21.26	21.65	21.56	21.49	β-Actin	**shC**	16.52	16.93	16.71	16.72
	**sh-ADGRG6-s**	23.9	23.82	23.96	23.89		**sh-ADGRG6-s2**	16.54	16.57	16.87	16.66
	**sh-ADGRG6-s**	23.69	23.54	23.7	23.64		**sh-ADGRG6-s1**	16.48	16.7	16.44	16.54

No.3		Ct	Ct	Ct				Ct	Ct	Ct	
ADGRG6	**shC**	21.38	21.66	21.43	21.49	β-Actin	**shC**	16.97	16.8	16.9	16.89
	**sh-ADGRG6-s**	24.15	24.04	23.75	23.98		**sh-ADGRG6-s2**	16.75	16.66	16.48	16.63
	**sh-ADGRG6-s**	23.7	23.5	23.83	23.67		**sh-ADGRG6-s1**	16.25	16.61	16.37	16.41

Overexpression ADGRG6 expression

priPC-1

No.1		Ct	Ct	Ct	**Mean value**			Ct	Ct	Ct	**Mean value**
ADGRG6	**Vec**	21.93	21.9	22.06	21.97	β-Actin	**Vec**	16.85	16.6	16.96	16.8
	**OE-ADGRG6**	20.2	20.47	20.33	20.33		**OE-ADGRG6**	18.33	18.23	18.21	18.26

No.2		Ct	Ct	Ct				Ct	Ct	Ct	
ADGRG6	**Vec**	22.57	22.55	22.74	22.62	β-Actin	**Vec**	17.86	17.96	17.78	17.87

	**OE-ADGRG6**	20.13	20.3	20.47	20.3		**OE-ADGRG6**	18.64	18.66	18.68	18.66

No.3		Ct	Ct	Ct				Ct	Ct	Ct	
ADGRG6	**Vec**	22.81	22.99	22.99	22.93	β-Actin	**Vec**	17.63	17.97	17.71	17.77
	**OE-ADGRG6**	20.4	20.34	20.28	20.34		**OE-ADGRG6**	18.64	18.4	18.79	18.61

PATU-8988

No.1		Ct	Ct	Ct	**Mean value**			Ct	Ct	Ct	**Mean value**
ADGRG6	**Vec**	22.82	22.84	22.77	22.81	β-Actin	**Vec**	17.97	17.61	17.9	17.83
	**OE-ADGRG6**	18.55	18.28	18.46	18.43		**OE-ADGRG6**	16.47	16.47	16.74	16.56

No.2		Ct	Ct	Ct				Ct	Ct	Ct	
ADGRG6	**Vec**	23.42	23.39	23.33	23.38	β-Actin	**Vec**	17.78	17.71	17.63	17.71
	**OE-ADGRG6**	18.85	18.88	18.66	18.8		**OE-ADGRG6**	16.82	16.57	16.42	16.6

No.3		Ct	Ct	Ct				Ct	Ct	Ct	
ADGRG6	**Vec**	21.22	21.32	21.37	21.31	β-Actin	**Vec**	17.72	17.87	18.02	17.87
	**OE-ADGRG6**	16.92	17.03	17.29	17.08		**OE-ADGRG6**	16.93	16.85	16.9	16.89
