# Supplementary figures and images for: Characterization of ADGRG6 as a potential molecular oncotarget of pancreatic cancer
Source: Cell Death Dis. 2026 May 6;17(1):604. doi: 10.1038/s41419-026-08766-2 (PMC13316050; doi:10.1038/s41419-026-08766-2)

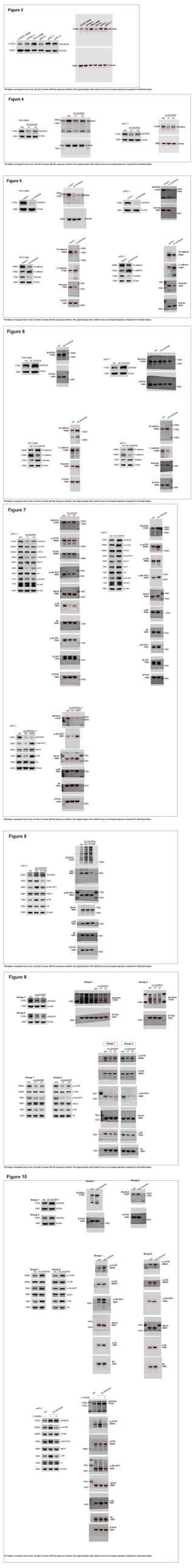

Supplement: Supplementary file 2 — original data of western bolt [file 41419_2026_8766_MOESM2_ESM.tif]
